# Improvement of epilepsy secondary to acquired immunodeficiency syndrome with intestinal microbiota preparations: a case report

**DOI:** 10.1186/s42494-024-00156-5

**Published:** 2024-04-15

**Authors:** Chuhui Lin, Ting Zeng, Yuhong Deng

**Affiliations:** 1 Department of Clinical Nutrition, the Second Affiliated Hospital of Guangzhou Medical University, Guangzhou, 510260 China; 2 Institute of Neuroscience and Department of Neurology, the Second Affiliated Hospital of Guangzhou Medical University, Guangzhou, 510260 China

**Keywords:** Acquired immunodefciency syndrome, Epilepsy, Bacteroides fragilis

## Abstract

**Background:**

Epilepsy secondary to acquired immunodeficiency syndrome (AIDS) can be challenging to manage. The potential interactions between antiretroviral drugs and antiepileptic drugs may result in the failure of both treatments. Therefore, it is crucial to develop more effective strategies to enhance the clinical outcomes of patients.

**Case presentation:**

We report a case of epilepsy secondary to AIDS. After administration of Bacteroides Fragilis 839 (BF839), the secondary generalized tonic-clonic seizures disappeared, the frequency of complex partial seizures decreased by 70%, and the duration of each episode was shortened. Additionally, long-term diarrhea associated with antiretroviral therapy for AIDS resolved, and the syphilis serofast reaction turned negative. No serious adverse reactions were observed during the three-year follow up.

**Conclusions:**

This case report suggests that the specific gut microbiota preparation could possibly improve refractory epilepsy in HIV patients while also potentially alleviating adverse reactions to antiretroviral drugs and concurrent syphilis infection. Our case may provide a new perspective for the treatment of HIV infection/AIDS.

## Background

Acquired immunodeficiency syndrome (AIDS) is a severe infectious disease caused by human immunodeficiency virus (HIV) infection, which primarily impairs the immune system and leads to cumulative damage in multiple systems. Epilepsy is highly prevalent in individuals with AIDS, with reported incidence rate ranging 6–11%. The management of AIDS-associated epilepsy often involves long-term administration of anti-seizure medications [1]. The potential interactions between antiretroviral drugs and anti-seizure medications are intricate and extensive, potentially leading to treatment failure of either antiviral or antiepileptic therapies [2]. In addition, a proportion of patients present with refractory epilepsy, which means that their seizures do not respond well to standard treatments. This highlights the need for novel strategies to improve the clinical outcomes of the patients.

Bacteroides fragilis 839 (BF839) is a non-toxic strain of Bacteroides fragilis [3] that exhibits immunomodulatory effects [4]. It has been available in the Chinese market for over 20 years [5] without any reported serious adverse reactions. Findings of our previous research indicate that when administered orally as an adjunctive therapy, BF839 had an effective rate of 61% in patients with refractory epilepsy who were unresponsive to three types of medication [6]. For patients with a recent diagnosis of “potentially autoimmune-related epilepsy”, the use of BF839 as the primary therapeutic approach achieved a seizure-freedom rate of 75% within one year [7], with no severe or long-term side effects reported.

The role of the gut microbiota in modulating immune function is widely acknowledged. Studies have provided evidence for the potential advantages of probiotics and prebiotics in the treatment of AIDS [8]. Supplementation with probiotics and prebiotics does not cause severe adverse reactions in AIDS patients with immune dysfunction [8].

Here, we report a case of epilepsy secondary to AIDS who showed significant reductions of seizures after BF839 treatment. In addition, the use of BF839 reduced the gastrointestinal side effects of antiretroviral drugs and had an efficacy against the coexisting syphilis. We also present a three-year follow up on this patient’s condition, in order to provide new ideas for clinical treatment.

## Case presentation

A 29-year-old Han Chinese male, who was an unmarried freelance worker, was admitted to the Second Affiliated Hospital of Guangzhou Medical University on August 2, 2020. The patient denied any family history of genetic disorders, epilepsy, AIDS, or syphilis. His parents were in good health conditions. Based on the Nutritional Risk Screening 2002, the patient exhibits a favorable nutritional status with no identified risks. Physical examination did not show any positive signs in the nervous system.

At the time of admission, the patient had been suffering from epilepsy for 9 years. In July 2014, during a comprehensive medical assessment, the patient was diagnosed with HIV-1 virus infection and treatment was immediately initiated with potent antiretroviral medications, including zidovudine tablets and lopinavir ritonavir tablets. The patient developed chronic diarrhea following the initiation of the antiretroviral therapy, with symptoms of yellowish, aqueous stools occurring 1–2 times daily, without accompanying abdominal discomfort.

**Fig. 1:**
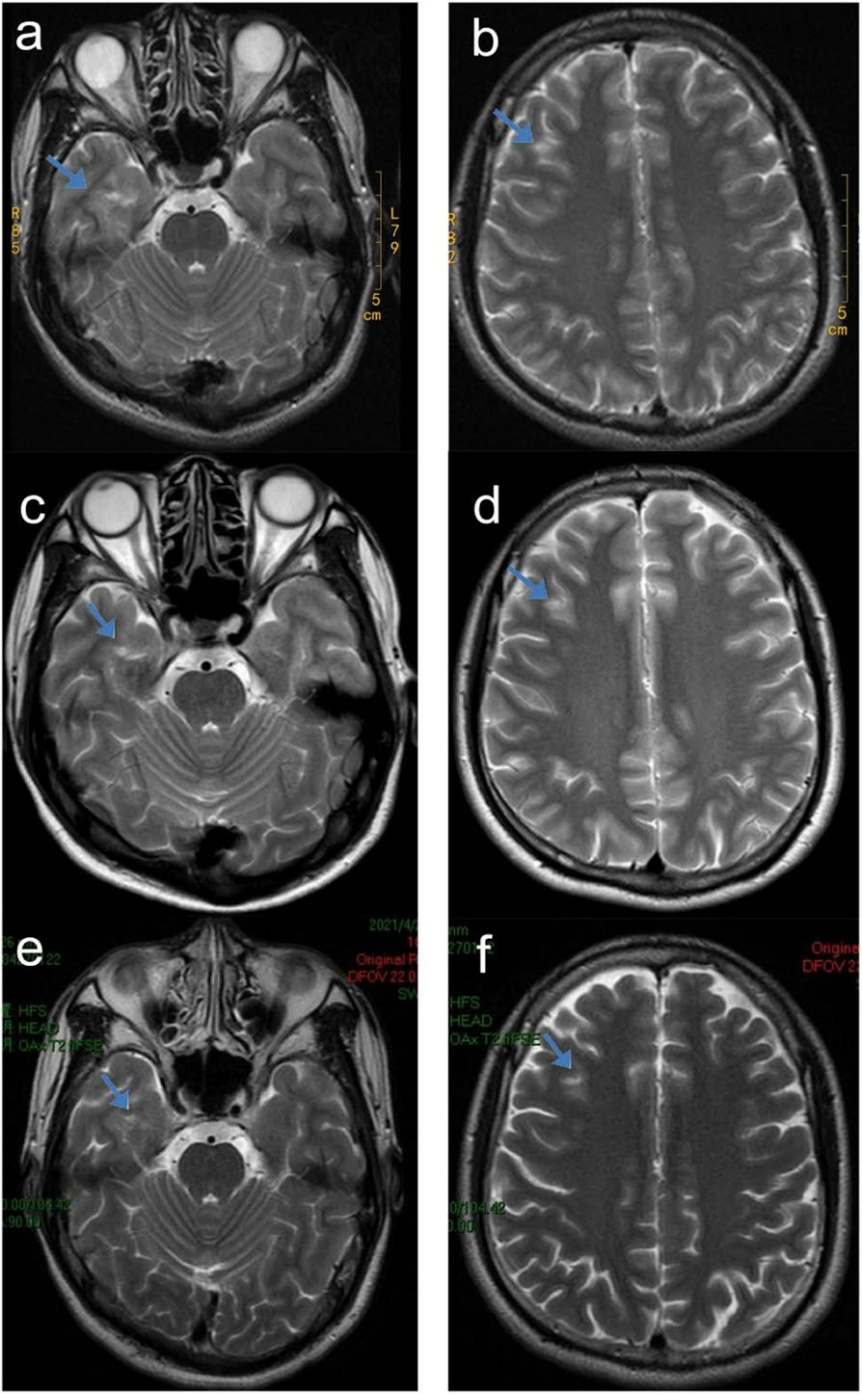
In August 2014, an abnormal patchy signal shadow was observed on the medial inferior side of the right temporal lobe. The shadow had an unclear boundary, and had a size of approximately 29 * 32 mm (**a**, arrow). Additionally, there was an abnormal signal shadow in the white matter of the right frontal lobe (**b**, arrow). In February 2020, abnormal patchy signal shadows were observed in the subcortical region of the anterior part of the right temporal lobe (**c**, arrow) and in the white matter of the right frontal lobe (**d**, arrow). In April 2021, abnormal patchy signal shadows were observed in the medial inferior region of the right temporal lobe (**e**, arrow) and in the white matter of the right frontal lobe (**f**, arrow)

**Fig. 2:**
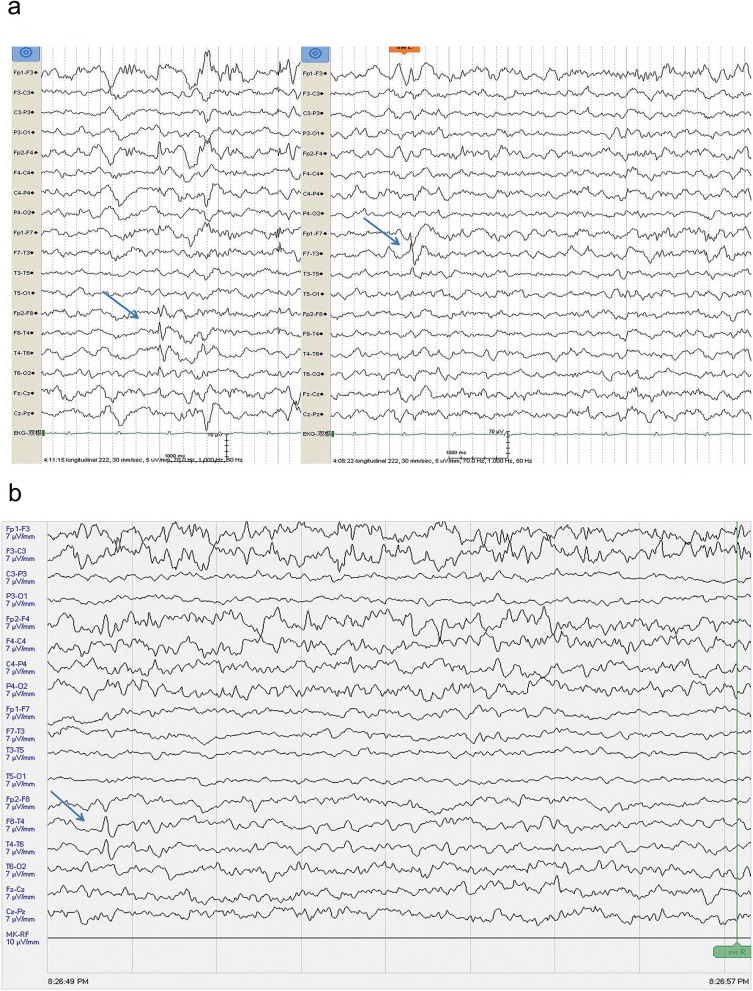
In March 2020, electroencephalograms (EEG) showed bilateral temporal lobe discharges during sleep (**a**, arrow). In March 2021, the EEG showed right temporal lobe discharges during sleep (**b**, arrow)

In 2014, the patient was additionally diagnosed with epilepsy following the occurrence of recurrent tonic-clonic seizures (characterized by loss of consciousness, limb convulsions, falling, with episode lasting for 3 min). The administration of lamotrigine failed to yield satisfactory results, leading to the introduction of levetiracetam, which resulted in the cessation of tonic-clonic seizures. However, the patient continued to experience complex partial seizures, characterized by a loss of consciousness accompanied by automatisms in the hand and the mouth. These seizures typically lasted for 2–3 min. The patient voluntarily discontinued antiepileptic medication in 2016, but resumed treatment in 2018 due to the recurrence of tonic-clonic seizures. After a 2-year treatment regimen involving monotherapy and combination therapy with medications such as lamotrigine, sodium valproate, levetiracetam, and clonazepam, the epilepsy remained uncontrolled. The patient experienced an average of 3–4 episodes of complex partial seizures per month, as well as 0–1 episode of tonic-clonic seizures per month. Brain magnetic resonance imaging (MRI) scans conducted in August 2014 and February 2020 revealed abnormal patchy signal shadows in the right temporal and frontal lobes (Fig. 1a–d). Electroencephalogram (EEG) performed in March 2020 exhibited bilateral temporal epileptiform discharges (Fig. 2a).

In 2015, syphilis infection was identified during a medical check-up, and the patient promptly received intramuscular benzylpenicillin injections. Subsequent to the treatment, the Treponema pallidum antigen serum test (TRUST) yielded negative results. In 2017, a recurrence of syphilis occurred, accompanied by a skin rash on the trunk resembling pityriasis rosea, and the TRUST titer increased to 1:64, leading to the diagnosis of secondary syphilis. The patient received treatment with ceftriaxone and doxycycline. Subsequently, the TRUST titer gradually decreased to 1:8 and remained at a low titer level for an extended period but did not reach a negative level. As of July 2020, the TRUST titer remained at 1:1, indicating a phenomenon known as syphilis serofast reaction.

In August 2020, Bacteroides fragilis BF839 (produced by Totem Life Medicine Research Co., Ltd., 10 g/pack, with a bacterial count of > 10^6^, administered at a dosage of 10 g once daily) was incorporated into the patient’s original treatment regimen, which comprised of 0.5 g sodium valproate twice daily, 0.5 g levetiracetam twice daily, and 1 mg clonazepam twice daily. Following the administration of BF839, the frequency of complex partial seizures of the patient decreased from 3–4 times per month to once per month, accompanied by a reduction in the severity of the seizures. Hand and mouth automatisms were not observed, with only transient episodes of impaired consciousness lasting for several seconds. Furthermore, secondary generalized tonic-clonic seizures were completely eliminated. In June 2021, the patient discontinued BF839 and experienced a secondary major tonic-clonic episode in August. Upon resuming BF839 in August 2021, the frequency of complex partial seizures reduced to once per month and remained stable for one year. However, from September 2022 to October 2023, the patient once again discontinued BF839, resulting in a gradual increase in the frequency of complex partial seizures to 3–4 times per month, without any tonic-clonic seizures. The addition of the new anti-seizure medication Lacoxamide in May 2023 was found to be ineffective. In April 2021, MRI still revealed abnormal patchy signal shadows in the right temporal and frontal lobes, but the area decreased compared to before (Fig. 1e, f). In March 2021, EEG recording showed that the discharges changed from bilateral to unilateral (Fig. 2b).

After one month of BF839 administration, the diarrhea caused by antiretroviral drugs completely resolved. The patient had bowel movements once every 1–2 days, with formed, normal brown color stools. The patient remained free of diarrhea for three years thereafter.

**Fig. 3:**
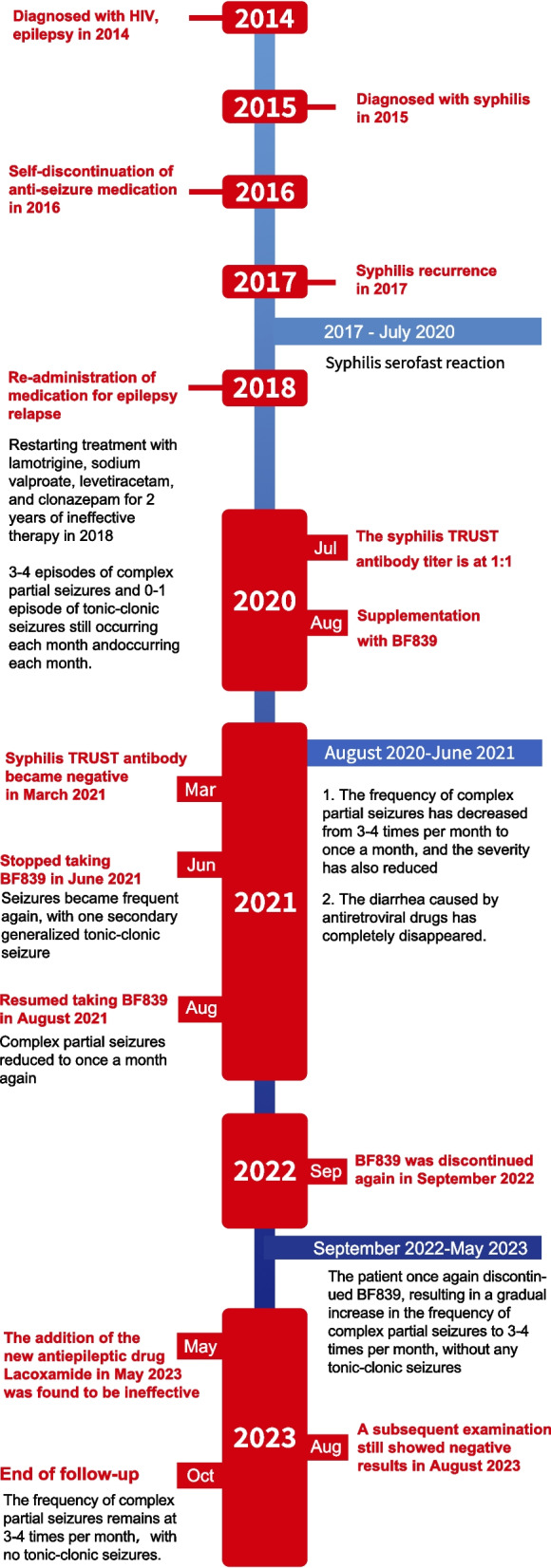
Treatment timeline of the case

In December 2020, the antiretroviral drug regimen was modified to incorporate tenofovir tablets, lamivudine tablets, and dolavirin tablets. Subsequently, in March 2022, the antiretroviral medication was further modified to dolavirin and lamivudine. In March 2021, a follow-up examination showed that the syphilis TRUST results turned negative. In August 2023, a subsequent examination still showed negative TRUST results. The timeline is illustrated in Fig. 3.

## Discussion

The treatment outcomes of the patient over the past three years consistently suggest that the addition of BF839 therapy effectively reduces epileptic seizures. It was noted that cessation of the therapy resulted in the recurrence of seizures to the initial state, whereas re-administration of BF839 reinstated their efficacy. This recurrent pattern suggests a potential therapeutic effect of BF839 in improving secondary epileptic seizures associated with HIV infection. It may also imply that a longer duration of treatment is required for oral administration of BF839, as premature discontinuation could potentially lead to a relapse of seizures. Importantly, throughout the three-year treatment period, the patient did not experience any significant adverse reactions, indicating the safety of BF839. Safety is a crucial factor to consider due to the long-term treatment option for HIV-associated epileptic seizures.

Based on the relationship between gut microbiota and immunity, the use of probiotics for immune reconstitution has become a hot topic in the treatment of HIV infection. Research has found that HIV infection leads to dysbiosis of the gut microbiota in patients, characterized by changes in microbial diversity, reduction of beneficial symbiotic bacteria, and an increase in potential pathogens [9]. Particularly, the dysbiosis manifests as a decrease of bacteria that play important roles in maintaining epithelial barrier health and immune regulation, as well as an increase of bacteria with pro-inflammatory potential [10–12]. HIV-infected patients exhibit a significant increase in the relative abundance of Prevotella and a decrease in the abundance of Bacteroides compared to the healthy controls [12–14]. Inflammatory factors such as tumor necrosis factor- α (TNF-α) and interleukin-6 (IL-6) are elevated in patients with HIV infection/AIDS, strongly suggesting a possible association between disease progression and inflammatory response [15]. Bacteroides fragilis has been found to reduce the levels of these inflammatory factors [16]. Bacteroides fragilis can regulate the conversion of CD4 + T cells to Foxp3 + regulatory T cells (Tregs) [17]. Tregs can secrete interleukin-10 (IL-10) to regulate the responses of Th17 lymphocytes and Th1 lymphocytes [18], and suppress the production of pro-inflammatory interleukin-17 (IL-17) by intestinal immune cells to prevent inflammatory bowel diseases [18]. Therefore, Bacteroides fragilis has an “anti-inflammatory” function, which may be one of the mechanisms for its effectiveness in this case.

HIV carriers often have concurrent syphilis infections. After receiving treatment of syphilis, the patients typically show negative results in the Treponema pallidum particle agglutination assay. However, some may exhibit persistently low levels of positive results, a phenomenon known as syphilis serofast reaction. It has been reported that about 34% of patients with syphilis remain serofast in China [19], which is mainly attributed to the immune responses. The serofast reaction rate is even higher among AIDS patients with compromised immune systems (40%) compared to the ordinary syphilis patients [20]. Finding ways to improve the serological fixation seroreversion rate in AIDS patients with concurrent syphilis is a clinical challenge. In this case, the patient showed seroreversion, which was maintained for 3 years after taking BF839 for six months, suggesting the potential of BF839 to improve the seroreversion rate. This effect may also be attributed to the previously mentioned immunomodulatory function of BF839.

AIDS patients require regular administration of a diverse array of antiretroviral medications. However, the prolonged usage of these drugs can give rise to a spectrum of adverse effects such as diarrhea. Studies have revealed that antiretroviral drugs per se possess the capacity to exacerbate dysbiosis within the intestinal microbiota [21, 22]. Consequently, modulation of gut microbial equilibrium is emerging as a promising avenue for mitigating the gastrointestinal sequelae associated with such therapeutic interventions. Currently, there is a lack of consensus regarding the precise therapeutic role of probiotics in ameliorating HIV-associated diarrhea [8]. Our case report provides evidence of the effectiveness of probiotics, which may be related to the use of different strains.

There is a limitation in this study. We did not collect patients’ fecal samples before initiation of the treatment and after the treatment to assess changes in the gut microbiota, which may otherwise provide evidence to elucidate the mechanism.

## Conclusions

This case report suggests that the specific gut microbiota preparation could possibly improve refractory epilepsy in HIV patients while also potentially alleviating adverse reactions to antiretroviral drugs and concurrent syphilis infection. This may provide a new perspective for the treatment of HIV/AIDS.

## Data Availability

All data generated or analyzed during this study are included in this published article.

## Abbreviations

AIDS: Acquired Immunodeficiency Syndrome
BF839: Bacteroides Fragilis 839
EEG: Electroencephalogram
HIV: Human immunodeficiency virus
MRI: Magnetic resonance imaging
TRUST: Treponema pallidum antigen serum test
